# Supplementary material for: Splice-Junction-Based Mapping of Alternative Isoforms in the Human Proteome
Source: Cell Rep. Author manuscript; Available in PMC 2020 Jan 15. (PMC6961840; doi:10.1016/j.celrep.2019.11.026)
Supplement: 2 [file NIHMS1546469-supplement-2.zip › DF1/PXD009737/TestisTrypsin/TestisTrypsin_65_IFT57_YINNQLENLVQEYRAAQAQLSEVMEELEK.pdf]

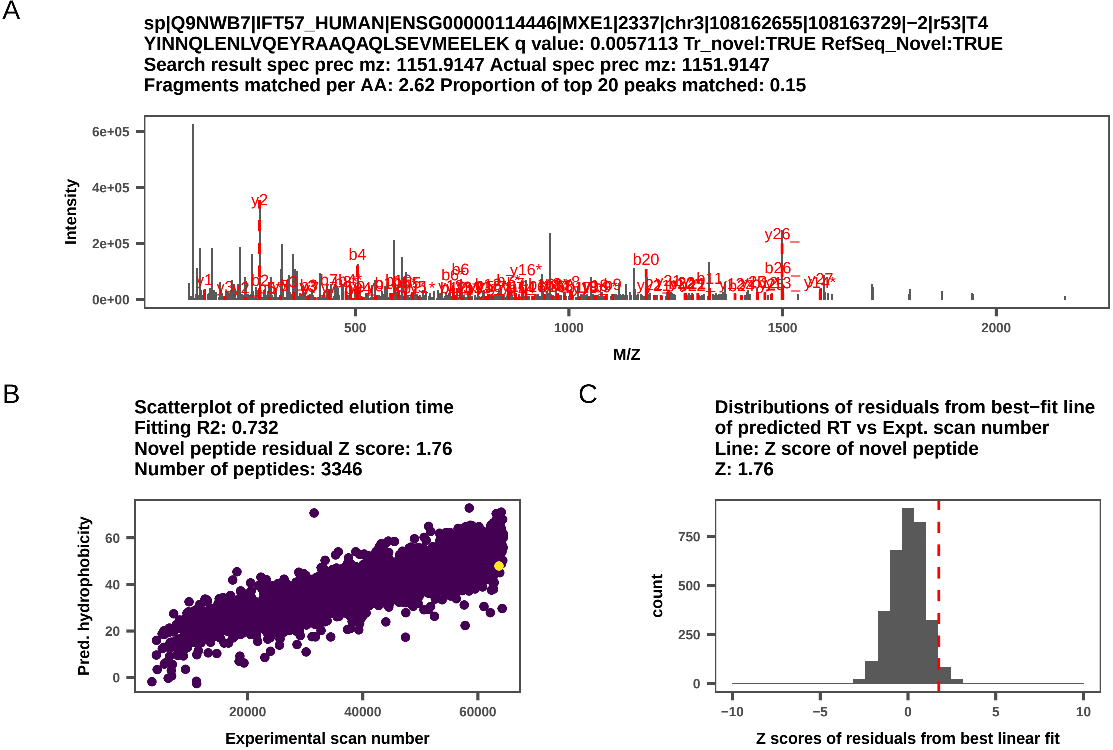
A
sp|Q9NWB7|IFT57_HUMAN|ENSG00000114446|MXE1|2337|chr3|108162655|108163729|−2|r53|T4
YINNQLENLVQEYRAAQAQLSEVMEELEK q value: 0.0057113 Tr_novel:TRUE RefSeq_Novel:TRUE
Search result spec prec mz: 1151.9147 Actual spec prec mz: 1151.9147
Fragments matched per AA: 2.62 Proportion of top 20 peaks matched: 0.15
Intensity
6e+05
4e+05
2e+05
0e+00
y2
b4
b6
y16*
b20
y26_
b26
y1
M/Z
500
1000
1500
2000
B
Scatterplot of predicted elution time
Fitting R2: 0.732
Novel peptide residual Z score: 1.76
Number of peptides: 3346
Pred. hydrophobicity
60
40
20
0
20000
40000
60000
Experimental scan number
C
Distributions of residuals from best−fit line
of predicted RT vs Expt. scan number
Line: Z score of novel peptide
Z: 1.76
count
750
500
250
0
−10
−5
0
5
10
Z scores of residuals from best linear fit
